# Efficacy of traditional Chinese medicine in treating severe pneumonia: A meta-analysis and systematic review

**DOI:** 10.1097/MD.0000000000048983

**Published:** 2026-05-22

**Authors:** Aoqiang Zhou, Haiqun Lin, Zhen Chen, Lina Luo, Xinyi Ye, Leng Li

**Affiliations:** a State Key Laboratory of Dampness Syndrome of Chinese Medicine, The Second Affiliated Hospital of Guangzhou University of Chinese Medicine, Guangzhou, Guangdong, China; b Department of Graduate School, Guangzhou University of Chinese Medicine, Guangzhou, Guangdong, China; c The Master Degree Application of Equivalent Educational Level of Guangzhou University of Chinese Medicine, Guangzhou, Guangdong, China; d Dongguan Hospital of Guangzhou University of Chinese Medicine, Dongguan, Guangdong, China.

**Keywords:** efficacy, severe pneumonia, traditional Chinese medicine

## Abstract

**Background:**

Severe pneumonia (SP) is a critical respiratory disease characterized by its severity and high mortality rate. Traditional Chinese medicine (TCM) has gradually been used as an adjunctive therapy in clinical treatment of SP, yet its efficacy and safety lack systematic evidence. This study aims to analyze, evaluate, and compare current research evidence on TCM adjunctive therapy for SP, providing a basis for clinical treatment of this condition.

**Methods:**

A systematic search was conducted on both English and Chinese online databases (PubMed, Embase, Cochrane Library, Web of Science, CNKI, Wanfang Data, VIP) to identify relevant papers published from inception to November 2023. High-quality randomized controlled tests (RCTs) were selected for analysis. Outcome measures related to SP were extracted from the literature. Statistical analysis was performed using RevMan 5.3 (Cochrane) software to assess heterogeneity among included studies, calculate mean differences, odds ratios, and their 95% confidence intervals, and detect publication bias.

**Results:**

A total of 34 RCTs met the inclusion criteria. Comparative analysis revealed that integrating TCM with conventional treatment significantly improves the overall clinical effective rate(*P* < .05). Specifically, it markedly alleviates TCM symptoms (*P* < .05). Additionally, TCM adjunctive therapy reduces levels of inflammatory markers including white blood cells, C-reactive protein (*P* < .05), and decreases Clinical Pulmonary Infection Score and Acute Physiology and Chronic Health Evaluation II scores compared to conventional treatment alone (*P* < .05). Compared to conventional treatment alone, TCM adjunctive therapy also enhances arterial blood gas indices (PaO_2_) (*P* < .05) and pulmonary function (forced expiratory volume in 1 second) (*P* < .05). Complication rates and mortality are lower in the TCM adjunctive therapy group(*P* < .05). Moreover, TCM treatment significantly boosts immune function (cluster of differentiation 4+ T lymphocyte) (*P* < .05).

**Conclusion:**

Integrating TCM with conventional treatment shows advantages in the efficacy of SP treatment, clinical symptoms management, inflammatory markers, and severity grading. Compared to conventional treatment alone, TCM adjunctive therapy improves immune function, pulmonary function, complication rates. However, future research should focus on large-scale, high-quality RCTs to further validate the effectiveness and safety of TCM adjunctive therapy for SP.

## 1. Introduction

Severe pneumonia (SP) is characterized by progressive inflammation of lung tissue, leading to rapid deterioration and potentially fatal outcomes such as respiratory failure and systemic inflammatory response.^[1]^ Its mortality rate ranges from 50 to 76%, making it a significant cause of infectious disease-related deaths worldwide.^[2,3]^ Clinical management primarily involves antimicrobial therapy, respiratory and circulatory support, nutritional support, and immune modulation. Early and accurate pathogen identification through microbiological diagnostics is crucial for guiding timely antibiotic treatment, which can significantly improve prognosis and reduce mortality.^[4,5]^ Despite symptom improvement, challenges persist in treatment due to antimicrobial resistance and inappropriate antibiotic use, resulting in difficulties, high costs, and poor outcomes.^[6,7]^

Traditional Chinese medicine (TCM) demonstrates unique advantages in treating SP, as studies indicate its significant roles in antimicrobial activity, immune modulation, and inflammation reduction.^[8]^ To further elucidate the clinical value of TCM in treating SP, this paper comprehensively compares TCM with conventional treatments and conducts a meta-analysis. It aims to provide evidence-based support for clinical practice.

## 2. Materials and methods

### 2.1. Inclusion and exclusion criteria

Inclusion criteria: study type: randomized controlled tests (RCTs); study participants: Patients diagnosed with SP; experimental group received conventional treatment plus TCM, while the control group received conventional treatment alone.

Exclusion criteria: review articles, case reports, conference papers, letters; literature for which full text cannot be obtained; duplicate publications; studies that do not include the observational indicators considered in this analysis; studies combining with other diseases.

### 2.2. Literature screening, quality assessment, and data extraction

Literature search was performed through PubMed, Web of science, Embase, Cochrane Library, CNKI, Wanfang Data, and VIP to retrieve literature from the establishment to November 1, 2023. The English search terms were: SP, RCT, random controlled test. The Chinese search terms were: SP, randomized, RCT, randomized controlled test.

All retrieved articles will be independently screened by 2 reviewers according to the inclusion and exclusion criteria, and relevant data will be extracted from articles that meet the criteria. If there are disagreements during the screening process, they will be resolved through discussion between the 2 reviewers or by consulting a third party. The extracted data from the literature mainly include: title, publication date, and authors of the paper; type of study design, design details, and risk of bias (including randomization method, blinding, allocation concealment, etc); sample size, age of patients, gender ratio, and interventions; study outcome measures. If some data are missing, the corresponding author may be contacted to obtain the missing information. The Cochrane criteria guidelines will be used to assess the risk of bias in RCTs, and the overall quality of the included studies will be evaluated based on the total score.

### 2.3. Statistical methods

Statistical analysis of the extracted observational indicators will be performed using Review Manager 5.3 software. The heterogeneity among the studies will be assessed using *P* values and *I*^2^ values. If *P* > .05 and *I*^2^ < 50%, it indicates no heterogeneity among the studies, and a fixed-effects model will be used. If *P* < .05 and *I*^2^ > 50%, it indicates significant heterogeneity, and a random-effects model will be used.

Continuous variables will be analyzed using mean difference (MD), while categorical variables will be analyzed using odds ratio (OR). All variables will be calculated with 95% confidence intervals (CI). The significance level for this study is *α* = 0.05, and a *P* value of < .05 will be considered statistically significant.

## 3. Results

**Figure 1.**
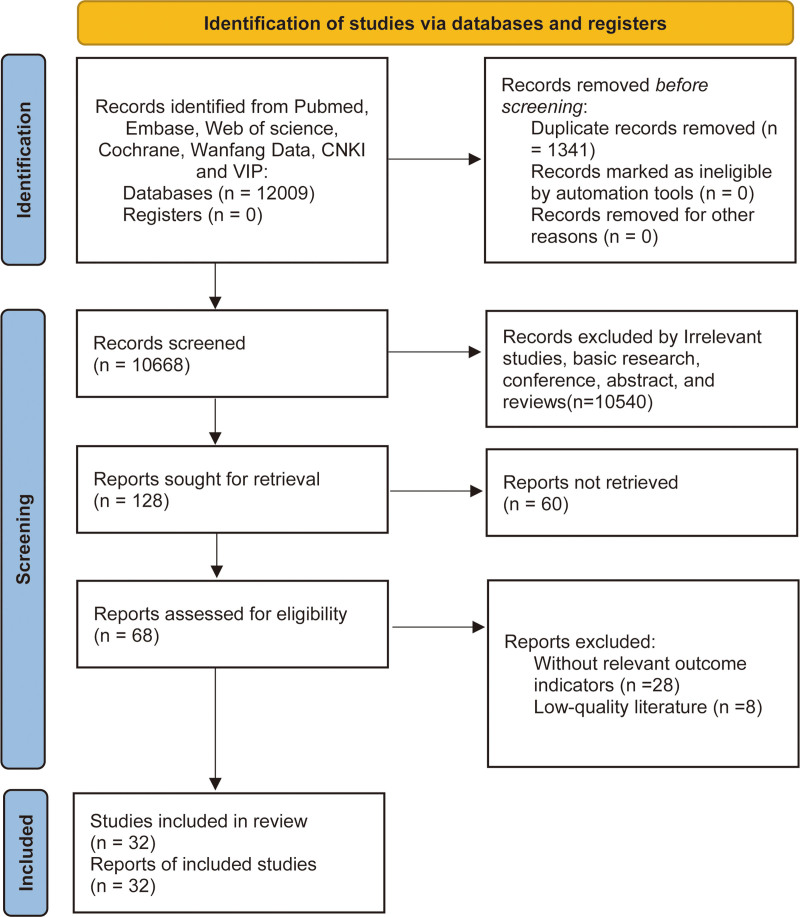
Flow diagram for selection of studies.

3.1. Literature inclusion results: A total of 12,009 articles were retrieved, and 32 articles were ultimately included.^[9–40]^ The inclusion process is shown in Figure 1.

**Figure 2.**
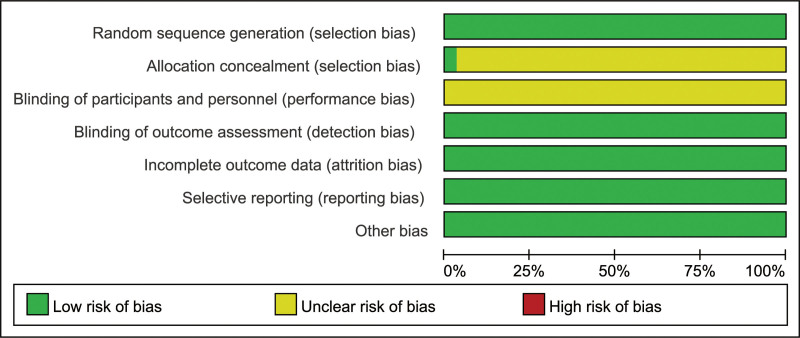
Risk of bias graph.

**Figure 3.**
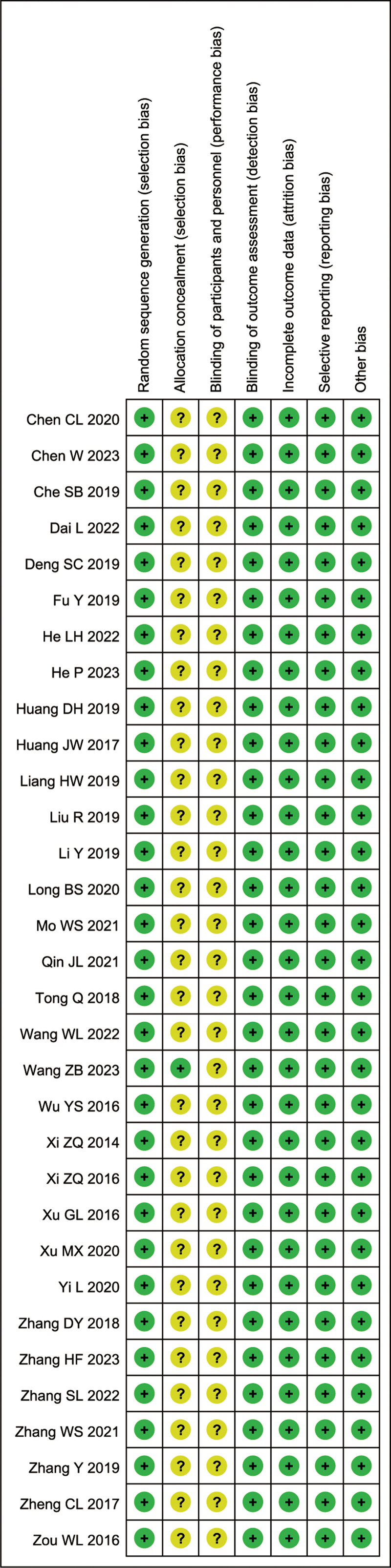
Risk of bias summary.

3.2. Basic characteristics and bias assessment results of included studies: The basic characteristics of the included studies are shown in Table 1, and the quality assessment results are shown in Figures 2 and 3. All of the included studies described the randomization method (Figs. 2 and 3).

**Table 1:** Basic characteristics of included studies.

Study	Publication Date	Case collection time	N (T/C)	Age (T/C)	Gender		Outcome indicators
					M (T/C)	F (T/C)	
Wang ZB 2023^[9]^	2023.1	2019.1–2020.1	30/30	44.43 ± 15.22/45.47 ± 14.02	16/17	14/13	④⑤⑩
Mo WS 2021^[10]^	2021.7	2018.2–2020.2	26/27	55.62 ± 4.5/55.24 ± 4.36	15/14	12/12	①⑥
Xu GL 2016^[11]^	2016.9	2014.3–2015.9	30/30	56/58	19/20	11/10	①③⑥
Chen W 2023^[12]^	2023.2	2019.6–2022.6	50/50	51.45 ± 9.87/51.32 ± 9.72	28/29	22/21	①⑤
Zou WL 2016^[13]^	2016.9	2013.10–2015.6	30/30	44.9 ± 5.7/45.2 ± 5.3	18/17	12/13	⑤⑥
Dai L 2022^[14]^	2022.7	2019.1–2020.1	55/55	67.32 ± 3.28/66.24 ± 3.19	30/28	25/27	⑤⑩
Fu Y 2019^[15]^	2019.10	2014.1–2017.1	45/45	55.12 ± 3.45/54.89 ± 3.29	24/22	21/23	①
Zhang DY 2018^[16]^	2018.11	2013.2–2014.7	45/45	59.9 ± 3.9/59.5 ± 3.8	27/26	18/19	①
Zheng CL 2017^[17]^	2017.9	2015.1–2016.6	48/48	68.27 ± 8.49/67.59 ± 7.32	25/27	23/21	①
Liu R 2019^[18]^	2019.4	2017.1–2018.4	49/49	66.49 ± 8.52/65.80 ± 8.14	28/25	21/24	⑥⑩
Chen CL 2020^[19]^	2020.7	2017.2–2018.10	52/52	42.61 ± 17.09/38.46 ± 16.52	29/32	23/20	④
Yi L 2020^[20]^	2020.4	2018.1–2019.6	37/37	61.3 ± 6.9/64.6 ± 7.5	23/21	14/16	②
Xu MX 2020^[21]^	2020.1	2016.9–2018.6	46/46	47.93 ± 5.14/47.87 ± 5.19	29/27	17/19	①⑦
He LH 2022^[22]^	2022.12	2020.8–2021.10	25/25	60.44 ± 5.33/59.45 ± 5.25	15/16	10/9	①⑤
Qin JL 2021^[23]^	2021.7	2016.12–2019.1	55/55	53.20 ± 11.78/53.12 ± 11.74	29/31	26/24	④
Wang WL 2022^[24]^	2022.1	2019.12–2021.1	42/42	56.51 ± 2.37/55.24 ± 2.32	23/26	19/16	①⑧
Huang DH 2019^[25]^	2019.11	2016.1–2018.12	53/53	59.26 ± 7.37/58.49 ± 7.53	29/31	24/22	①④⑧
Long BS 2020^[26]^	2020.8	2018.2–2019.5	55/55	70.23 ± 8.16/69.45 ± 7.12	35/32	20/23	⑦⑩
Xi ZQ 2014^[27]^	2014.11	2013.1–2014.1	40/20	72.25 ± 16.17/72.05 ± 16.36	24/9	16/11	⑥
Zhang WS 2021^[28]^	2021.10	2018.6–2019.12	29/29	61.69 ± 5.75/63.07 ± 7.61	16/18	13/11	③
Liang HW 2019^[29]^	2019.1	2017.1–2017.12	30/30	52.47 ± 9.25/48.17 ± 8.56	14/18	16/12	③
Zhang SL 2022^[30]^	2022.4	2020.1–2021.1	40/40	51.12 ± 2.26/50.99 ± 2.24	24/22	26/18	⑨
He P 2023^[31]^	2023.6	2020.8–2022.3	47/47	65.36 ± 2.04/64.96 ± 1.91	25/23	22/24	①③⑤⑥⑩
Zhang Y 2019^[32]^	2019.2	2016.1–2017.12	30/30	65.37 ± 7.35/54.85 ± 7.42	18/16	12/14	⑤⑥
Deng SC 2019^[33]^	2019.8	2015.8–2018.12	41/41	53.27 ± 5.18/52.94 ± 5.22	24/25	17/16	①
Che SB 2019^[34]^	2019.2	2016.10–2018.2	47/47	56.71 ± 4.06/55.29 ± 4.18	26/25	21/22	①③
Xi ZQ 2016^[35]^	2016.5	2013.6–2014.6	30/30	72.25 ± 16.165/72.05 ± 16.356	26/14	14/6	①②③⑤⑥
Tong Q 2018^[36]^	2018.2	2014.1–2017.1	52/52	65.24 ± 2.7/65.13 ± 2.24	31/33	21/19	①④
Zhang HF 2023^[37]^	2023.7	2020.1–2021.3	43/43	58.42 ± 2.60/57.58 ± 2.67	25/23	18/20	①②⑦⑩
Wu YS 2016^[38]^	2016.4	2014.5–2015.10	32/32	66.79 ± 2.38/64.37 ± 1.49	19/21	13/11	②
Li Y 2019^[39]^	2019.6	2018.1–2018.12	38/38	57.86 ± 7.31/58.45 ± 7.56	19/20	19/18	⑨
Huang JW 2017^[40]^	2017.1	2014.3–2015.12	55/55	62.9 ± 5.48/62.75 ± 5.42	33/31	22/24	①⑩

C = control group, CD4^+^ = cluster of differentiation 4+ T lymphocyte, F = female, FEV1 = forced expiratory volume in 1 second, M = male, N = number of patients.T = test group.

① Efficacy Rate; ② Traditional Chinese Medicine Symptom Scores; ③ APACHE II; ④ CPIS; ⑤ CRP; ⑥ WBC; ⑦ PaO2_;_ ⑧ CD4^+^⑨ FEV1; ⑩ Complications.

### 3.1. Meta-analysis results

#### 3.1.1. Efficacy rate

**Figure 4.**
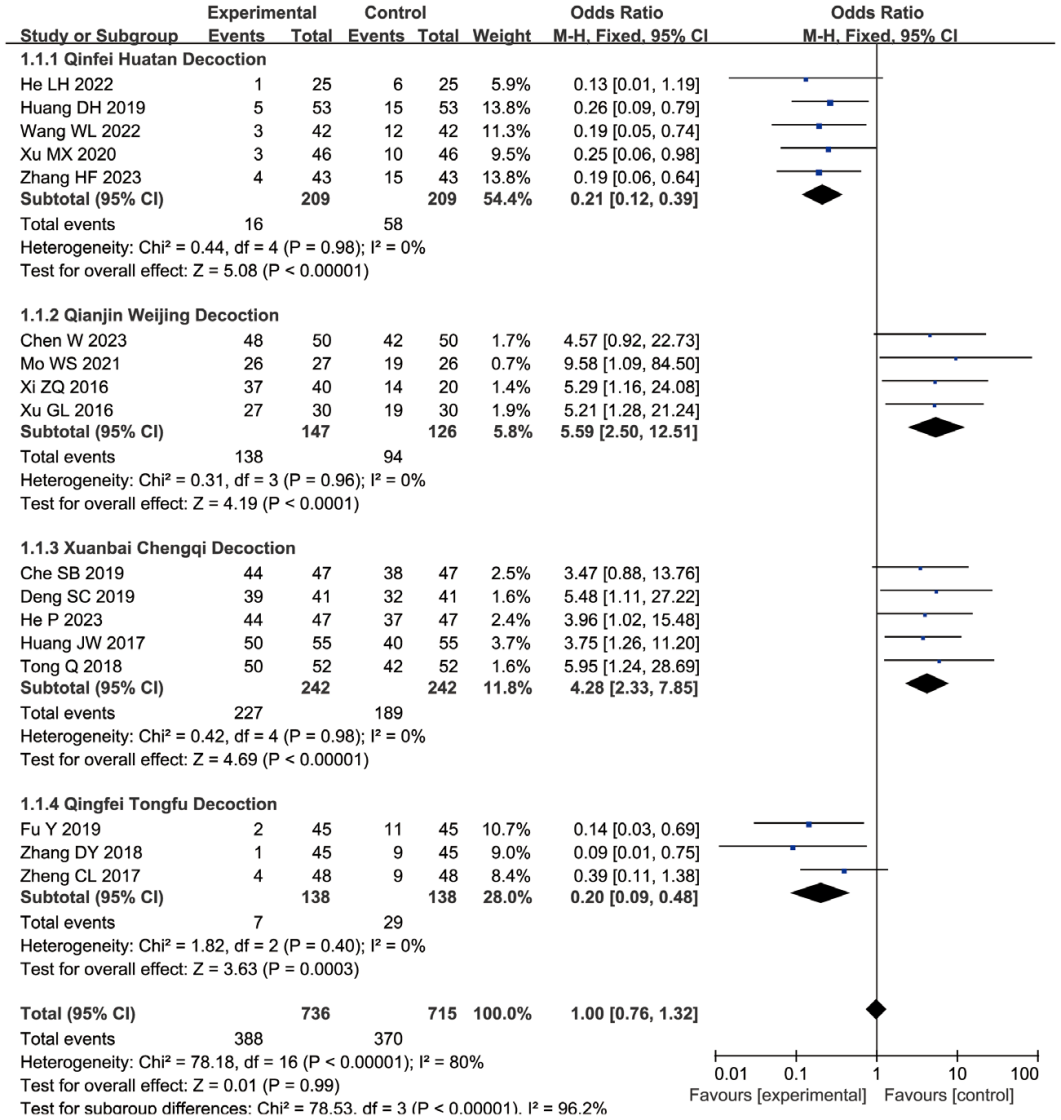
Differences in the efficacy rate of TCM combined with conventional treatment versus conventional treatment alone. TCM = traditional Chinese medicine.

A total of 17 articles were included for this measure (combined treatment group: 736 cases, conventional treatment group: 715 cases). In the subgroup analysis, 5 articles used Qingfei Huatan Decoction in the experimental group, 4 articles used Qianjin Weijing Decoction, 5 articles used Xuanbai Chengqi Decoction, and the remaining 3 articles used Qingfei Tongfu Decoction. The analysis of the 4 subgroups showed *I*^2^ = 0%, indicating the use of a fixed-effects model. The difference of Qingfei Decoction (OR = 0.21, 95% CI = [0.12 0.39], *P* < .00001), Qianjin Weijing Decoction (OR = 5.59, 95% CI = [2.50 12.51], *P* < .00001), Xuanbai Chengqi Decoction (OR = 4.28, 95% CI = [2.33 7.58], *P* < .00001), and Qingfei Tongfu Decoction (OR = 0.2, 95% CI = [0.09 1.32], *P* < .00001) were statistically significant between the combined treatment group and conventional treatment group. The combined treatment group had a higher efficacy rate, as shown in Figure 4.

#### 3.1.2. TCM symptom scores

An analysis of 4 studies compared the TCM combined with combined treatment and conventional treatment alone (combined treatment group: 152 cases, conventional treatment group: 132 cases) in terms of TCM Symptom Scores in the treatment of SP: 2 on Qinfei Huatan Decoction, 2 on Qianjin Weijing Decoction.

**Figure 5.**
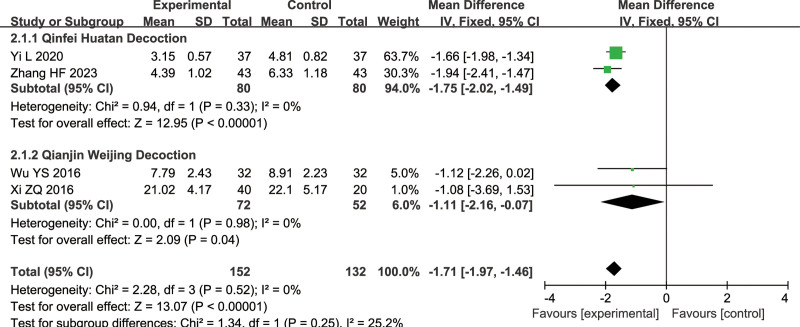
Differences in the TCM symptom scores of TCM combined with conventional treatment versus conventional treatment alone. TCM = traditional Chinese medicine.

The analysis of 2 subgroups showed no heterogeneity (Qinfei Huatan Decoction: *I*^2^ = 0%, Qianjin Weijing Decoction: *I*^2^ = 0%), indicating the use of a fixed-effects model. There was a significant difference between the combined treatment group and conventional treatment group (Qinfei Huatan Decoction: MD = −1.75, 95% CI = [−2.02, −1.49], *Z* = 12.95, *P* < .00001, Qianjin Weijing Decoction: MD = −1.11, 95% CI = [−2.16, −0.07], *Z* = 2.09, *P* = .04), with the combined treatment group having a lower total TCM symptom score compared to the conventional treatment group, as shown in Figure 5.

#### 3.1.3. Acute Physiology and Chronic Health Evaluation (APACHE II)

**Figure 6.**
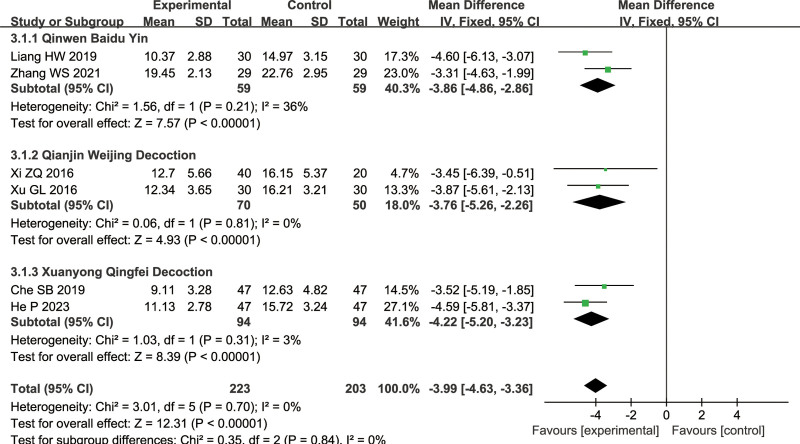
Differences in the APACHE II of TCM combined with conventional treatment versus conventional treatment alone. APACHE II = Acute Physiology and Chronic Health Evaluation, TCM = traditional Chinese medicine.

A total of 6 articles were included for this measure (combined treatment group: 223 cases, conventional treatment group: 203 cases): 2 on Qinwen Baidu Yin, 2 on Qianjin Weijing Decoction, 2 on Xuanyong Qingfei Decoction. The analysis of subgroups showed *I*^2^ < 50%, indicating the use of a fixed-effects model. There was a significant difference between the combined treatment group and conventional treatment group (Qinwen Baidu Yin: MD = −3.86, 95% CI = [−4.86, −2.86], *Z* = 7.57, *P* < .00001, Qianjin Weijing Decoction: MD = −3.76, 95% CI = [−5.26, −2.26], Z = 4.93, *P* < .00001, Xuanyong Qingfei Decoction: MD = −4.22, 95% CI = [−5.20, −3.23], Z = 8.39, *P* < .00001), with the combined treatment group having a lower APACHE II score, as shown in Figure 6.

#### 3.1.4. Clinical Pulmonary Infection Score (CPIS)

**Figure 7.**
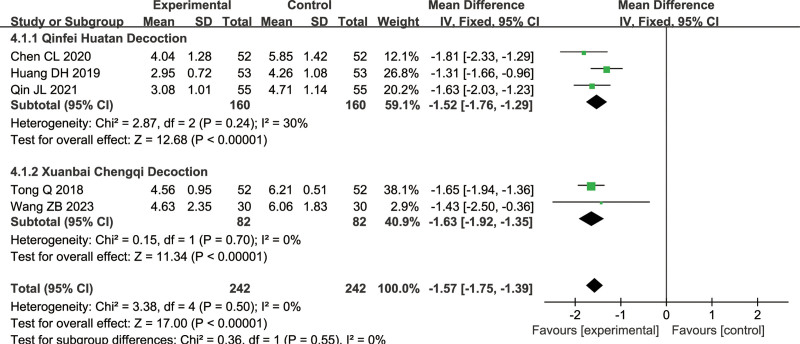
Differences in the CPIS of TCM combined with conventional treatment versus conventional treatment alone. CPIS = Clinical Pulmonary Infection Score, TCM = traditional Chinese medicine.

A total of 5 articles were included for this measure (combined treatment group: 242 cases, conventional treatment group: 242 cases): 3 on Qinfei Huatan Decoction, 2 on Xuanbai Chengqi Decoction. Since there was no significant heterogeneity (Qinfei Huatan Decoction: *I*^2^ = 30%, Xuanbai Chengqi Decoction: *I*^2^ = 0%). A fixed-effect model was used for the meta-analysis. The results showed that there was a significant difference between the combined treatment group and conventional treatment group (Qinfei Huatan Decoction: MD = −1.52, 95% CI = [−1.76, −1.29], Z = 12.68, *P* < .00001, Xuanbai Chengqi Decoction: MD = −1.63, 95% CI = [−1.92, −1.35], Z = 11.34, *P* < .00001), with the combined treatment group having a lower CPIS score, as illustrated in Figure 7.

#### 3.1.5. High-sensitivity C-reactive protein (hs-CRP)

**Figure 8.**
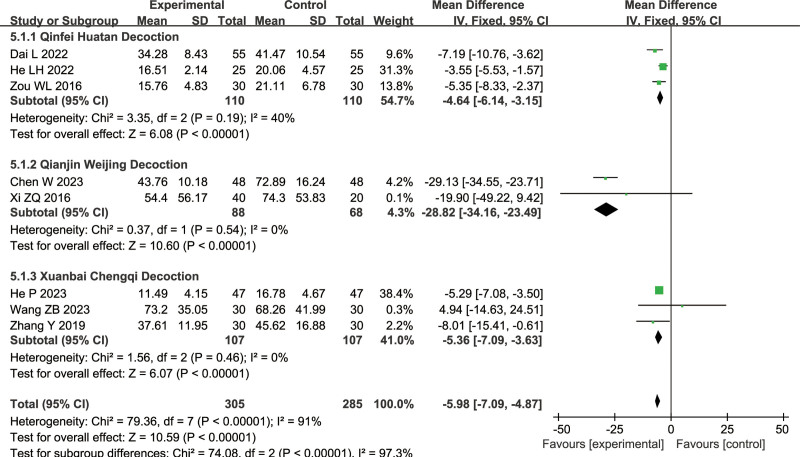
Differences in the hs-CRP of TCM combined with conventional treatment versus conventional treatment alone. hs-CRP = high-sensitivity C-reactive protein, TCM = traditional Chinese medicine.

Eight articles were included for hs-CRP analysis (combined treatment group: 305 cases, conventional treatment group: 285 cases): 3 on Qinfei Huatan Decoction, 2 on Qianjin Weijing Decoction, 3 on Xuanbai Chengqi Decoction. There was no heterogeneity in this analysis (Qinfei Huatan Decoction: *I*^2^ = 40%, Qianjin Weijing Decoction: *I*^2^ = 0%, Xuanbai Chengqi Decoction: *I*^2^ = 0%), indicating the use of a fixed-effects model. There was a significant difference between the combined treatment group and conventional treatment group (Qinfei Huatan Decoction: MD = −4.64, 95% CI = [−6.14, −3.15], *P* < .00001, Z = 6.08, Qianjin Weijing Decoction: MD = −28.82, 95% CI = [−34.16, −23.49], Z = 10.60, *P* < .00001, Xuanbai Chengqi Decoction: MD = −5.36, 95% CI = [−7.09, −3.63], Z = 6.07, *P* < .00001), with the combined treatment group having lower hs-CRP levels, as shown in Figure 8.

#### 3.1.6. White blood cell (WBC)

**Figure 9.**
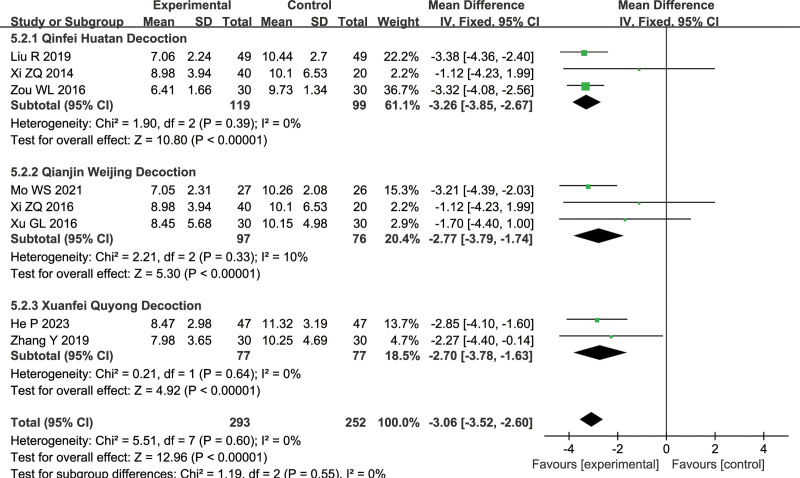
Differences in the WBC analysis of TCM combined with conventional treatment versus conventional treatment alone. TCM = traditional Chinese medicine, WBC = white blood cell.

An evaluation of WBC was conducted by 8 studies (combined treatment group: 293 cases, conventional treatment group: 252 cases): 3 on Qinfei Huatan Decoction, 3 on Qianjin Weijing Decoction, 2 on Xuanfei Quyong Decoction. There was no heterogeneity in this analysis (Qinfei Huatan Decoction: *I*^2^ = 0%, Qianjin Weijing Decoction: *I*^2^ = 10%, Xuanbai Chengqi Decoction: *I*^2^ = 0%), indicating the use of a fixed-effects model, indicating the use of a fixed-effects model. There was a significant difference between the combined treatment group and conventional treatment group (Qinfei Huatan Decoction: MD = −3.26, 95% CI = [−3.85, −2.67], *P* < .00001, Z = 10.80, Qianjin Weijing Decoction: MD = −2.77, 95% CI = [−3.79, −1.74], Z = 5.30, *P* < .00001, Xuanbai Chengqi Decoction: MD = −2.70, 95% CI = [−3.78, −1.63], Z = 4.92, *P* < .00001), with the combined treatment group having lower WBC levels, as shown in Figure 9.

#### 3.1.7. Blood gas analysis

### PaO_2_:

**Figure 10.**
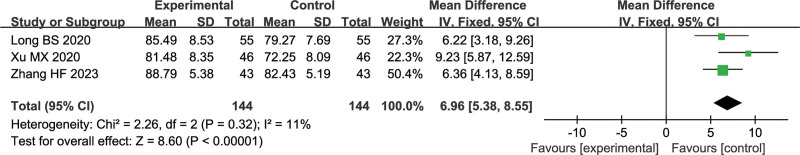
Differences in the PaO_2_ of TCM combined with conventional treatment versus conventional treatment alone. TCM = traditional Chinese medicine.

3 articles were included for PaO_2_ analysis (combined treatment group: 144 cases, conventional treatment group: 144 cases). The analysis showed *I*^2^ = 11%, indicating the use of a fixed-effects model. There was a significant difference between the 2 groups (MD = 6.96, 95% CI = [5.38, 8.55], *P* < .00001), with the combined treatment group having higher PaO_2_ levels, as shown in Figure 10.

#### 3.1.8. Immune factors

### Cluster of differentiation 4+ T lymphocyte (CD4^+^):

**Figure 11.**
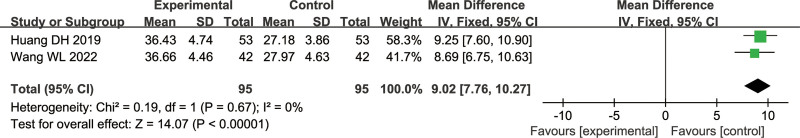
Differences in the CD4^+^ of TCM combined with conventional treatment versus conventional treatment alone. CD4^+^ = cluster of differentiation 4+ T lymphocyte, TCM = traditional Chinese medicine.

2 articles were included for CD4^+^ analysis (combined treatment group: 95 cases, conventional treatment group: 95 cases). The analysis showed *I*^2^ = 0%, indicating the use of a fixed-effects model. There was a significant difference between the 2 groups (MD = 9.02, 95% CI = [7.76, 10.27], *P* < .00001), with the combined treatment group having higher CD4^+^ levels, as shown in Figure 11.

#### 3.1.9. Pulmonary function indicators

### Forced expiratory volume in 1 second (FEV1):

**Figure 12.**
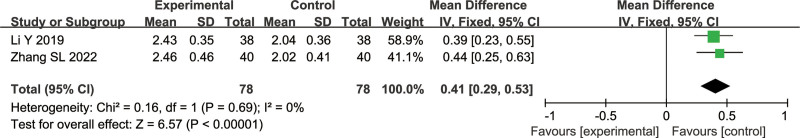
Differences in the FEV1 of TCM combined with conventional treatment versus conventional treatment alone. FEV1 = forced expiratory volume in 1 second, TCM = traditional Chinese medicine.

2 articles were included for FEV1 analysis (combined treatment group: 78 cases, conventional treatment group: 78 cases). The analysis showed *I*^2^ = 0%, indicating the use of a fixed-effects model. There was a significant difference between the 2 groups (MD = 0.41, 95% CI = [0.29, 0.53], *P* < .00001), with the combined treatment group having higher FEV1 levels, as shown in Figure 12.

#### 3.1.10. Complications

**Figure 13.**
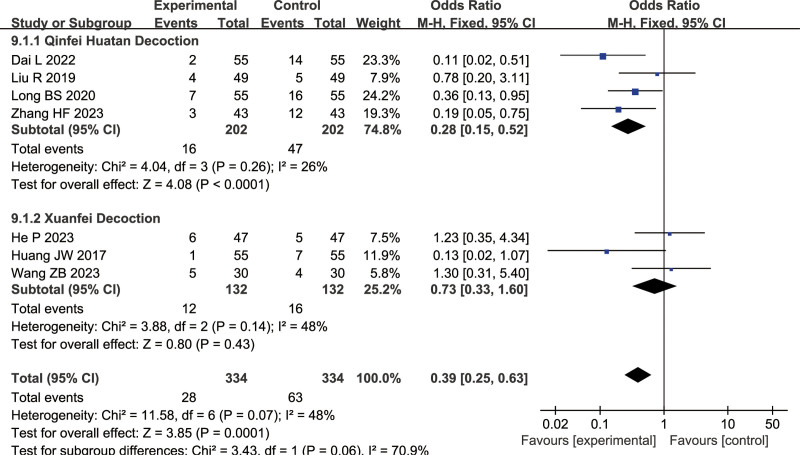
Differences in the complications of TCM combined with conventional treatment versus conventional treatment alone. TCM = traditional Chinese medicine.

Seven articles were included for complications analysis (combined treatment group: 334 cases, conventional treatment group: 334 cases): 5 on Qinfei Huatan Decoction, 3 on Xuanfei Decoction. The heterogeneity results of the 2 subgroups are shown as follows: Qinfei Huatan Decoction: *I*^2^ = 26%, Xuanfei Decoction: *I*^2^ = 48%, indicating the use of a fixed-effects model. There was a significant difference between the combined treatment group and conventional treatment group (Qinfei Huatan Decoction: OR = 0.28, 95% CI = [0.15, 0.52], Z = 4.08, *P* < .00001, Xuanbai Chengqi Decoction: OR = 0.73, 95% CI = [0.33, 1.60], Z = 0.80, *P* < .00001), with fewer complications observed in the combined treatment group, as shown in Figure 13.

#### 3.3.10. Publication bias

**Figure 14.**
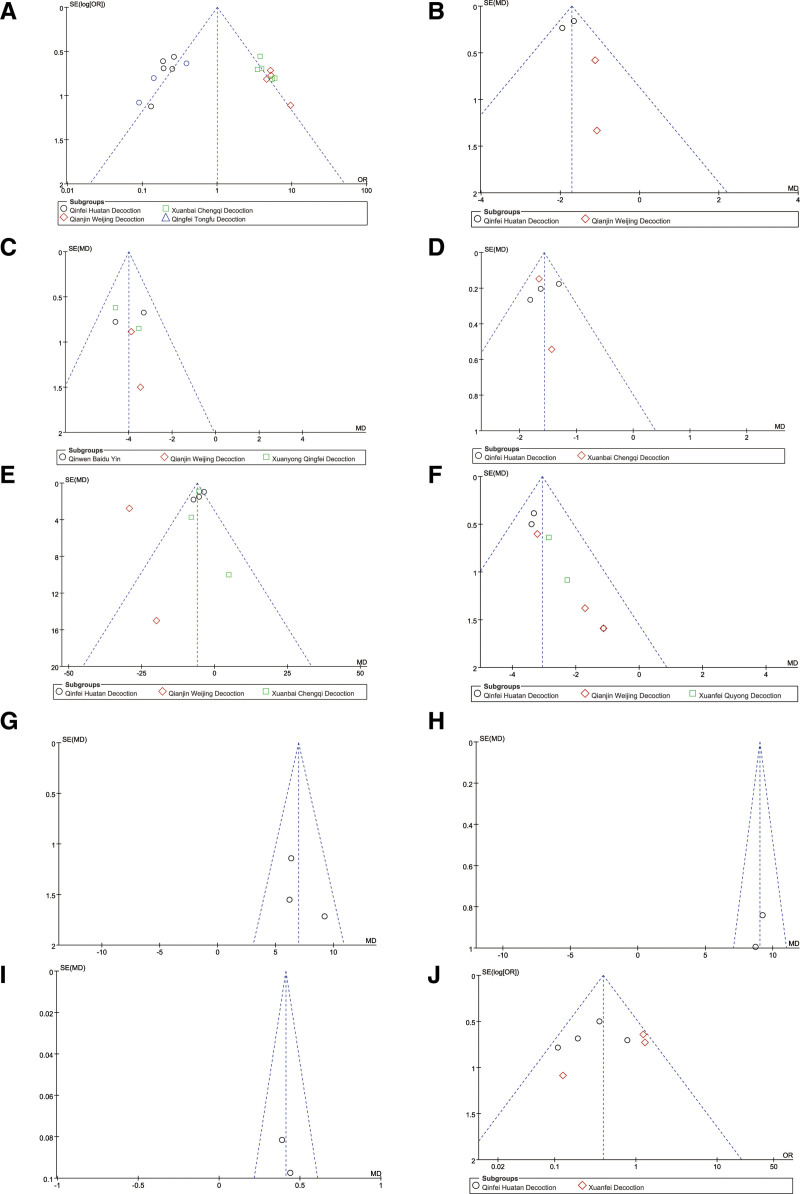
Funnel plot for detecting publication bias for studies comparing the effect of TCM combined with conventional treatment versus conventional treatment alone in the treatment of severe pneumonia. (A) efficacy rate; (B) TCM symptom scores; (C) APACHE II; (D) CPIS; (E) hs-CRP; (F) WBC; (G) PaO_2_; (H) CD4^+^; (I) FEV1; (J) complications. APACHE II = Acute Physiology and Chronic Health Evaluation, CD4^+^ = cluster of differentiation 4+ T lymphocyte, CPIS = Clinical Pulmonary Infection Score, FEV1 = forced expiratory volume in 1 second, hs-CRP = high-sensitivity C-reactive protein, TCM = traditional Chinese medicine, WBC = white blood cell.

Publication bias assessment was conducted for all outcome measures. The results indicated no significant publication bias, as shown in Figure 14.

## 4. Discussion

SP is often characterized by severe inflammatory conditions in the lung interstitium and alveoli caused by pathogens such as viruses, bacteria, and others.^[41–43]^ It progresses rapidly with urgent onset.^[44]^ SP can lead to high levels of inflammatory factors in lung tissues, triggering systemic inflammation, which may result in sepsis. This condition is often accompanied by hypotension, acute respiratory failure, shock, and severe hypoxemia.^[45]^ Approximately 5 to 10% of hospitalized community-acquired pneumonia (CAP) patients require intensive care unit admission due to respiratory failure and/or septic shock. Even among SP patients admitted to the intensive care unit, short-term mortality rates can still be as high as 30 to 60%, comprising 5 to 10% of all hospitalized CAP patients.^[46,47]^ In addition to its high incidence and mortality rates, pneumonia is one of the costliest diseases requiring hospitalization and remains a leading cause of death among infants in low-income countries and elderly individuals in high-income countries.

Although progress has been made in rapid diagnostic tests, new treatment regimens, and vaccine strategies, the mortality rate associated with SP remains a major concern, particularly among the elderly.^[48–51]^

Timely, accurate, and empirical treatment is crucial for reducing mortality rates. Antibiotic therapy remains the primary approach to combating infections, but the rise of antibiotic-resistant strains and the emergence of new pathogens and viruses pose greater challenges to treatment. Therefore, developing effective adjunct therapies is essential for addressing the high mortality rates of SP.^[52,53]^

TCM has been widely used in the treatment of chronic diseases, yet its application in critical illness remains less widely accepted and recognized. Therefore, conducting research on TCM treatment for SP is of significant importance for both treating pneumonia and promoting the use of TCM therapies.

SP in TCM falls within the category of “Shang Han” and “Wen Bing.” According to the “Nanjing” (Classic of Difficulties), this disease belongs to the broader category of “Shang Han.” In the “Wen Bing Jing Wei” (Synopsis of Warm Disease), it is described that during its onset, pathogenic factors such as wind-heat invade the lung wei (defensive qi), leading to heat affecting nutrient blood, qi, and the lung channels. The main symptoms include cough, sputum production, fever, dyspnea, and wheezing.

The pathological mechanisms involve the congelation of fluids into phlegm, stasis of blood forming stasis, initial invasion by pathogenic qi, and the mutual congealment of phlegm and stasis. Based on the theory of “phlegm and stasis,” the core pathogenesis of this disease is identified as blood stasis, phlegm obstruction, and qi deficiency.

TCM treatment of SP offers unique advantages, focusing on regulating qi, transforming phlegm, clearing heat, detoxifying, and nourishing qi and blood. In this study, the included prescriptions such as Qianjin Weijing Decoction and Maxing Shigan Decoction demonstrate significant efficacy in clearing lung-wei heat toxins. Other formulas like Qingjin Huatan Decoction are effective in resolving phlegm, while Qingjin Zhuyu Decoction not only clears heat but also resolves stasis.

Chinese herbal decoctions leverage the multifaceted actions of Chinese herbs to not only exhibit anti-inflammatory effects, relieve cough, and improve lung lesions but also to regulate the function of the body’s immune system.^[54]^

Empirical and targeted antibiotic therapy, glucocorticoids, mechanical ventilation, and fluid support are conventional treatments for severe CAP.^[55]^ However, up to 30% of patients still experience treatment failure, with a high mortality rate of up to 50%. Furthermore, even with symptomatic relief from conventional treatment, it often leads to various complications. In 2019, pneumonia caused nearly 2.5 million deaths globally, ranking it as the fourth leading cause of death worldwide.^[56,57]^

SP clinically presents with symptoms typical of acute infection or acute respiratory disease, including fever or hypothermia, leukocytosis or leukopenia, cough or sputum production, chest pain, dyspnea, rapid breathing, and potentially respiratory failure.^[58]^

The APACHE II score is commonly used to grade the severity of the disease. It serves as an important prognostic tool for predicting the outcome of SP patients to some extent, and is crucial in predicting mortality rates of severe illnesses. The CPIS serves as an alternative clinical diagnostic tool for SP and is an early clinical predictor of SP.^[59]^

The decrease in SP score and inflammatory biomarkers indicates a reduction in inflammation, which can predict an increase in survival rate of SP patients in clinical practice and indicate a good prognosis for SP. It also means the relief of symptoms and can help guide doctors in the use of antibiotics.^[60–62]^

Measurements of biomarkers in plasma can aid in the diagnosis of SP and guide initial antibiotic therapy, as well as assist in de-escalation and duration of antibiotic treatment. Key biomarkers include C-reactive protein, procalcitonin, interleukin-6, tumor necrosis factor-α, WBC, among others.^[63–65]^ A wide array of immune biomarkers can also be used to diagnose or predict SP infections, making them parameters for immune monitoring. These biomarkers include CD4^+^ T cells, CD8^+^ T cells, CD4^+^/CD8^+^ ratio, and others.^[66]^ TCM combined with conventional treatments can to some extent reduce biomarkers such as inflammatory and immune factors.

T cells are significantly reduced in patients with SP, which also leads to decreased immune function, poorer prognosis, and higher mortality rate. However, after treatment, there is a significant increase in T cells, which may help control the deterioration of the condition and improve clinical outcomes.^[67]^

FEV1 and forced vital capacity (FVC) are reliable indicators for assessing lung function impairment in patients with CAP, with significant decreases observed in most pneumonia patients’ FEV1 and FVC values.^[68,69]^ Reduced lung function also correlates with corresponding decreases in blood gas parameters. Measurement of arterial blood gas-related indicators is a common clinical vital sign management and monitoring technique, involving various monitoring parameters. Typical among these are PaO_2_, PaCO_2_, PaO_2_/FiO_2_, which directly reflect the efficiency of pulmonary gas exchange in patients, and are important monitoring indicators for assessing respiratory and blood pH balance disturbances. Monitoring these parameters can directly reveal residual imbalances such as respiratory CO_2_ retention in patients with pulmonary diseases.^[70]^

Rebound in FEV1 and FVC implies an improvement in the patient lung function and an improvement in the patient’s asthmatic dyspnea symptoms.^[71]^ The rebound of blood gas parameters is clinically independently correlated with the patient’s length of hospital stay, and to some extent may guide the patient’s discharge.^[72]^

This article comprehensively integrates multiple studies and conducts meta-analysis. By comparing several indicators, it is found that TCM treatment for SP has shown good efficacy. It can improve patient symptoms to a certain extent, alleviate inflammation, enhance immunity, and result in fewer complications compared to conventional treatments alone. Moreover, there is a significant reduction in mortality rate. These findings are consistent with some previous research results.^[73,74]^

## 5. Limitations

Moreover, in practice, the combination of TCM with conventional treatment significantly alleviates the economic burden on patients. While the therapeutic advantages of TCM in treating SP have been recognized, its shortcomings also need equal attention. Firstly, the essence of TCM lies in syndrome differentiation, which guides the treatment of SP under the guidance of TCM syndrome differentiation. Secondly, current research on TCM intervention for SP remains weak. Clinical studies lack sufficient emphasis on methodological rigor, with issues such as low sample sizes, few RCTs, poor reproducibility, and inappropriate selection of efficacy indicators, which affect the reliability and validity of research conclusions. The distinctive features and advantages of TCM therapy may not be fully realized, potentially leading to increased bias among studies and influencing research outcomes.

## 6. Conclusion

In summary, the combination of TCM with conventional treatment effectively enhances clinical efficacy, alleviates symptoms, and improves patients’ quality of life in the treatment of SP. This approach holds significant value for broad clinical application and promotion. Therefore, future efforts should focus on conducting high-quality, large-sample clinical studies, establishing integrated Chinese and Western medicine treatment plans and efficacy evaluation systems based on TCM syndrome differentiation, formulating clinical guidelines for integrated Chinese and Western medicine treatment of SP, and advancing the development of TCM therapy for critical and severe illnesses. Simultaneously, in-depth mechanistic research should be conducted around disease pathogenesis, treatment mechanisms, effective targets, etc, based on clinically effective TCM prescriptions. This will provide scientific foundations for the clinical application of TCM in treating critical illnesses, promote TCM theoretical innovation, and facilitate the development of new Chinese medicines.

## Acknowledgments

This research was financially supported by Dongguan Science and Technology of Social Development Program (20221800906122).

## Author contributions

**Conceptualization:** Leng Li.

**Data curation:** Leng Li.

**Writing – original draft:** Aoqiang Zhou, Haiqun Lin, Zhen Chen.

**Writing – review & editing:** Lina Luo, Xinyi Ye.
